# An Enhanced Rehabilitation Approach for a Patient With Leukemia and Severe Intensive Care Unit-Acquired Weakness Following Critical Pneumonia

**DOI:** 10.7759/cureus.84187

**Published:** 2025-05-15

**Authors:** Makoto Onji, Takanori Ohta, Shinji Kakizoe

**Affiliations:** 1 Department of Rehabilitation, Kitakyushu Municipal Medical Center, Kitakyushu, JPN; 2 Department of Hematology, Kitakyushu Municipal Medical Center, Kitakyushu, JPN

**Keywords:** aspiration pneumonia, enhanced rehabilitation, immunodeficiency, intensive care unit-acquired weakness, leukemia, mechanical ventilation, muscle strength, pancytopenia

## Abstract

Intensive care unit-acquired weakness (ICU-AW) is common in patients with sepsis, multiple organ failure, or prolonged mechanical ventilation; however, reports of ICU-AW during periods of immunodeficiency and cytopenia associated with leukemia treatment are limited. This report describes a case of a patient with leukemia who developed severe ICU-AW and was successfully managed with enhanced rehabilitation that progressively increased in both intensity and frequency. A female patient in her late 50s, diagnosed with acute myeloid leukemia and undergoing postremission therapy, developed aspiration pneumonia and required mechanical ventilation. She exhibited severe ICU-AW after extubation. After enhanced rehabilitation, including standing and walking exercises and strength training based on her clinical indicators, significant improvements in muscle strength and activities of daily living were achieved. She was discharged 72 days after extubation with no severe adverse events during rehabilitation. This report suggests that enhanced rehabilitation may be effective and safe in patients with severe ICU-AW who have immunodeficiency and pancytopenia.

## Introduction

Intensive care unit-acquired weakness (ICU-AW) refers to “a wide variety of disorders characterized by acute onset of neuromuscular impairment without any plausible cause other than critical illness, greater than that resulting from being bedridden for a prolonged period, and typically associated with multiorgan failure [1].” ICU-AW is systemic muscle weakness that occurs following critical illness and can be diagnosed using measures such as the Medical Research Council (MRC) sum score (by assessing manual muscle strength testing in 12 bilateral muscle groups. Each muscle group is scored from 0 to 5, resulting in a maximum possible total score of 60 points) [1]. This condition occurs in approximately 50% of patients with sepsis, multiple organ failure, or prolonged mechanical ventilation [2]. Current rehabilitation guidelines for critically ill patients recommend enhanced rehabilitation following ICU discharge [3]; however, a standardized consensus on the optimal frequency and specific rehabilitation protocols remains undefined. While numerous studies have demonstrated the effectiveness of exercise therapy interventions in patients with ICU-AW [4], evidence regarding outcomes in patients with hematologic malignancies and severe ICU-AW remains limited. This report describes a case of profound ICU-AW (MRC score: 0) in a leukemic patient with pancytopenia who achieved independent home discharge through enhanced rehabilitation, demonstrating the safety and efficacy of this approach even during high-risk hematological vulnerability.

## Case presentation

Patient's clinical course leading to the current admission

A woman in her late 50s (height: 155 cm; body mass index: 19.6 kg/m^2^), employed in the hospitality industry, was initially healthy without comorbidities. She had experienced low-grade fever, dyspnea, and decreased appetite for several days before visiting a local hospital. Initial blood tests revealed marked leukocytosis (white blood cell count: 120,000/μL), prompting her referral to our institution. Upon evaluation, she was diagnosed with FMS-like tyrosine kinase 3 mutation-positive acute myeloid leukemia (AML) with maturation. She received remission induction therapy with daunorubicin, cytarabine, and quizartinib, and hematological remission was confirmed. She then underwent postremission therapy with cytarabine and quizartinib. She was readmitted for a second course of postremission therapy.

Physical therapy program and physical functional status at hospital admission

Rehabilitation commenced upon hospital admission and consisted of two 40-minute sessions, five times per week, conducted in a rehabilitation room. The training protocol primarily included aerobic exercise (bicycle ergometer) and resistance training (weighted exercises, sit-to-stand exercises, squats, etc.) prescribed at a perceived exertion level of 13-15. Physical function was assessed by measuring exercise tolerance, grip strength, knee extensor strength, skeletal muscle index, and phase angle at both admission and discharge.　Exercise tolerance was measured using the six-minute walk test. The skeletal muscle index, muscle mass, and phase angle were assessed using a body composition analyzer (InBody 770; InBody Japan, Tokyo, Japan). Grip strength was measured with a Jamar-type hydraulic grip dynamometer (SH5001; Sakai Medical, Tokyo, Japan), while knee extensor strength was assessed using a handheld dynamometer (μTas MT1; ANIMA, Tokyo, Japan). The measurement results are listed in Table 1 [5-8].

**Table 1:** Changes in physical performance parameters during hospitalization The table shows the changes in the patient's physical performance between hospital admission and discharge. Although none of the parameters fully recovered to preadmission levels, the patient achieved independent activities of daily living by the time of discharge BW: body weight; BMI: body mass index; 6 MWD: six-minute walking distance; SMI: skeletal muscle mass index; Rt: right; Lt: left

Parameter	Hospitalization	Discharge	Reference range
BW (kg)	47.1	43.1	55.2 ± 9.2 (50-59 years)
BMI (cm/m^2^)	19.6	17.9	18.5-24.9
6 MWD (m)	385	210	400
Knee strength (kgf/BW) (Rt/Lt)	0.22/0.23	0.10/0.13	0.59 ± 0.12 (50-59 years)
Grip strength (kg) (Rt/Lt)	15.2/13.4	14.5/14.3	18
SMI (kg/m^2^)	5.3	4.8	<5.7

Clinical course from admission to tracheal intubation and during artificial management

**Figure 1:**
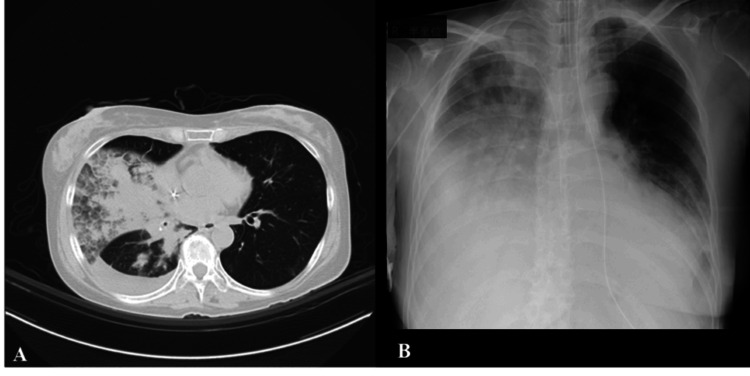
Imaging findings at intensive care unit admission. (A) CT findings. (B) Chest radiograph. Infiltrative shadows are observed throughout most of the right lung field CT: computed tomography

**Figure 2:**
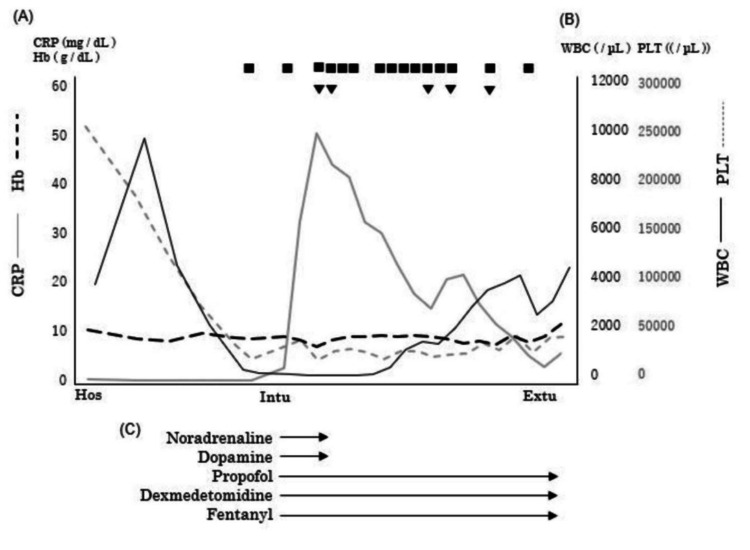
Changes in blood parameters from hospital admission to extubation (A) Serial change in CRP and Hb (B) Serial changes in WBC and PLT (C) Medication timeline. The timing of mechanical ventilation initiation for severe pneumonia coincided with the period of cytopenia. Arrows at the bottom highlight critical drug interventions Transfusion: ■: platelet concentrate; ▼: red cell concentrate CRP: C-reactive protein; Hb: hemoglobin; WBC: white blood cell; PLT: platelet; Hos: hospitalization; Intu: intubation; Extu: extubation

On admission, the patient had a performance status of 1 and was in good physical condition, with no decline in activities of daily living (ADL). She received postremission therapy with cytarabine and oral quizartinib, and then entered the cytopenic phase. Thirteen days after the start of the treatment, during the cytopenic phase (white blood cell count, 30/μL; platelet count, 23,000/μL), she experienced nausea followed by sudden vomiting, massive aspiration, and respiratory failure, necessitating intubation and mechanical ventilation. Figure 1 shows the imaging findings. She developed sepsis and remained hemodynamically unstable, requiring noradrenaline and dopamine for seven days. Deep sedation was achieved with propofol, dexmedetomidine, and fentanyl (Richmond Agitation-Sedation Scale score -4 to -5; see Figure 2). Total parenteral nutrition began on the fourth day of intubation. At onset, no neurological findings were suggestive of central nervous system disease (e.g., stroke, spinal cord injury, or neuromuscular disease), including motor or sensory disturbances, pupillary irregularities, or rigidity of the terminus, consistent with a clinical course of respiratory failure due to aspiration pneumonia.

Physical therapy program during mechanical ventilation

The physiotherapy continued postintubation using joint range-of-motion exercises and an electric ergometer (Escargot, PBE-1; Minato Medical Science Co., Osaka, Japan) to prevent limb contracture and disuse. In conjunction with nursing, the patient was positioned in left lateral decubitus and prone (from 16:00 to 9:00 the next morning) to improve the ventilation-perfusion ratio and enhance oxygenation at the lesion site. Except during daytime care and radiographic examinations, head-up exercises were carefully performed to optimize ventilation while closely monitoring the patient's circulatory status.

Enhanced rehabilitation approach

**Figure 3:**
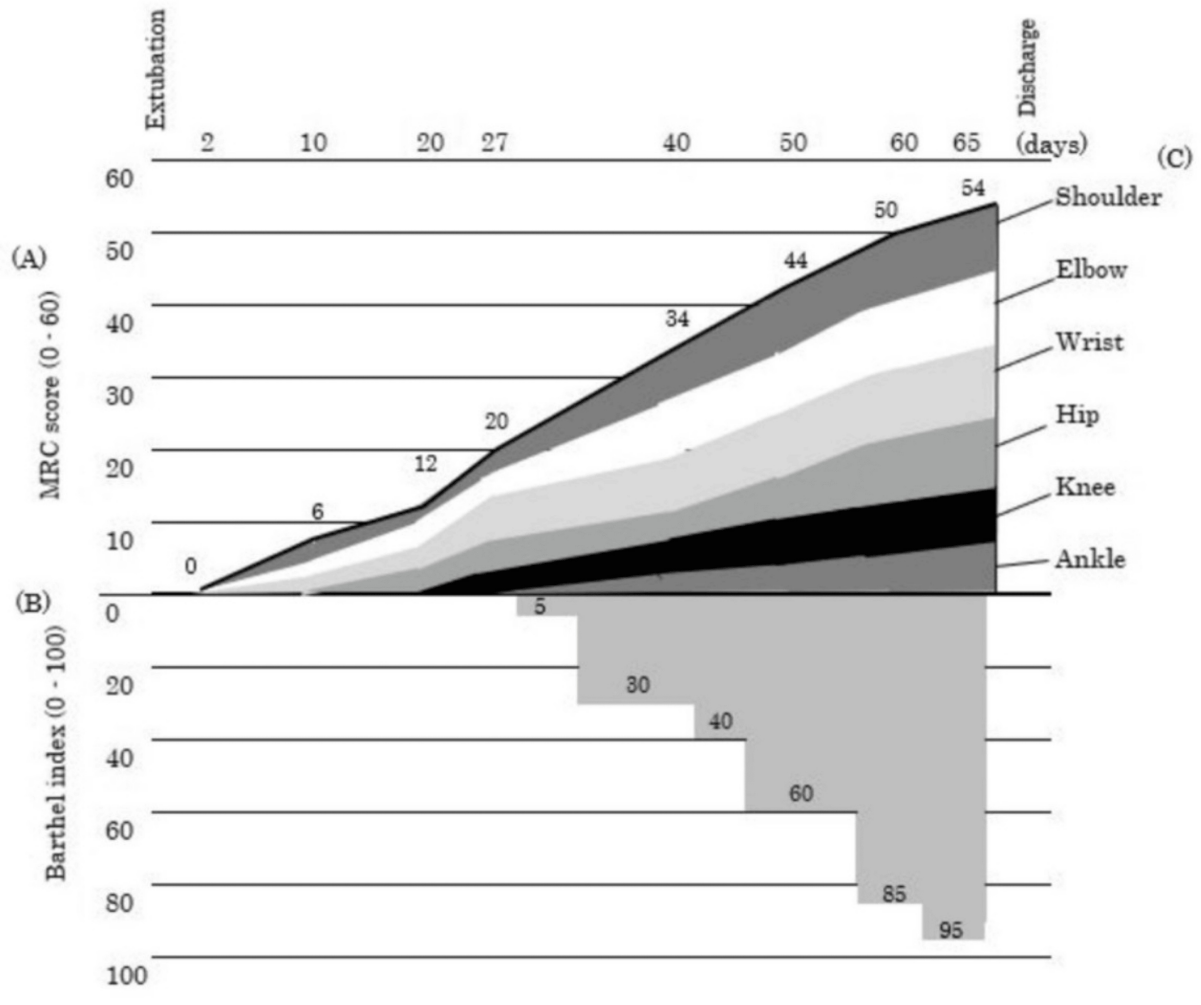
Temporal changes and details of MRC score and BI after extubation (A) Total MRC score (B) Barthel index (C) Serial changes in MRC scores by joint MRC score: 0 = no contraction, 5 = normal power (total score: 12 joint × 5 points) The X-axis shows actual assessment days after admission. Uneven intervals reflect real clinical follow-up timing MRC: Medical Research Council; BI: Barthel index

**Figure 4:**
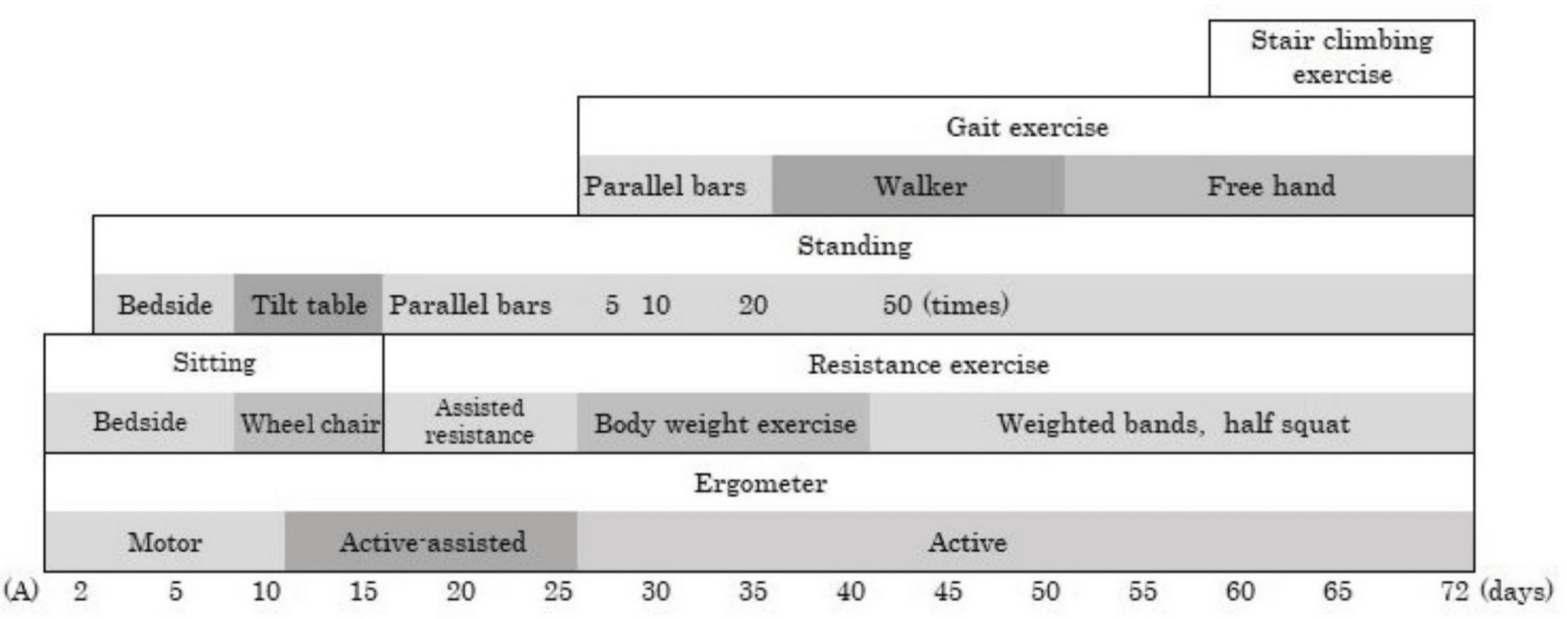
Rehabilitation timeline postextubation (A) Clinical timeline from extubation to discharge The upper section of the frame describes key rehabilitation programs, while the lower section details the means and methods. Numerical values for standing exercise indicate the number of repetitions ICU: intensive care unit

**Figure 5:**
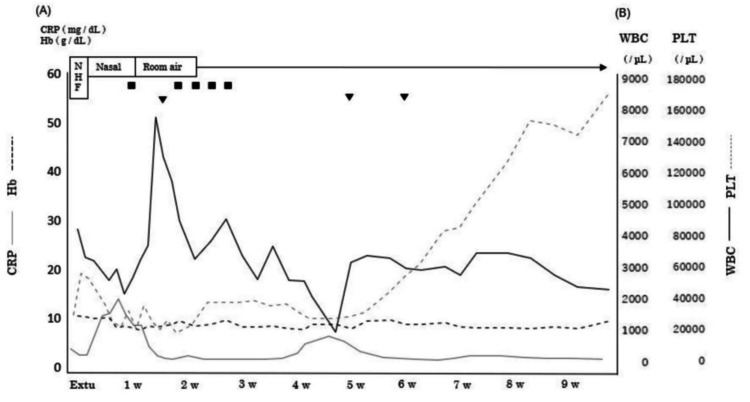
Changes in blood parameters during enhanced rehabilitation (A) Serial change in CRP and Hb (B) Serial changes in WBC and PLT Platelet counts showed gradual recovery over time. No supplemental oxygen was required beyond seven days postextubation Transfusion: ■: platelet concentrate; ▼: red cell concentrate NHF: nasal high flow; CRP: C-reactive protein; Hb: hemoglobin; WBC: white blood cell; PLT: platelet; Extu: extubation

The duration of tracheal intubation was 18 days. The hematological findings until extubation are shown in Figure 2. After extubation, her level of consciousness was normal, with no observed anisocoria, abnormal light reflexes, impaired eye movements, or facial nerve palsy. Subsequently, she exhibited flaccid muscle weakness in all four limbs, with an MRC score of 0 and diminished deep tendon reflexes. Head computed tomography findings also showed no abnormalities explaining the muscle weakness. Based on the clinical course and pathophysiological findings, including the MRC score, she was diagnosed with ICU-AW. Three days postventilator weaning, the patient was transferred to the general ward. After extubation, a speech therapist initiated swallowing exercises. Initially, the patient had aspiration episodes due to impaired swallowing function, which improved over time. Three days later, a swallowing diet was introduced, and the dietary consistency gradually improved. Their nutritional status remained between 4.0 and 5.9 g/dL for total protein and 2.4-3.3 g/dL for albumin during the exercise therapy period. The intervention involved one to two sessions of 40-60 minutes, performed seven days per week. The patient received 1-2 L/minute of oxygen via nasal cannula for the first seven days postventilator weaning. She also experienced considerable difficulty with head and trunk control. Despite stable respiratory and circulatory status, exercise tolerance was significantly reduced, leading to session discontinuation after approximately five minutes due to fatigue. An electric ergometer session was initiated with the patient in the supine position for 10 minutes. Ten days later, she was transferred to a wheelchair with full assistance. Thirteen days later, exercise therapy began in the rehabilitation room. The tilt table was strategically selected to safely promote orthostatic tolerance while minimizing the risk of hemodynamic instability in this critically ill patient. After 18 days, she practiced standing on parallel bars. The upper and lower limbs were stabilized using bands and weights, and full assistance was provided for standing and maintaining an upright position five times per session. Standing exercises were implemented in a carefully graded manner, with incremental increases in frequency tailored to the patient's daily tolerance levels to optimize lower extremity strengthening. A floor ergometer (Magnetic Bike Mini; Alinco, Osaka, Japan) was set at load level 1. The physical therapist provided manual support for five sessions at 20 rpm, with rest until the patient’s fatigue subsided and their heart rate returned to baseline. Initially, the load was based on revolutions but was later adjusted to continuous exercise duration, starting at three minutes and eventually extending to 15 minutes. This approach aimed to preserve muscle function and accelerate ADL recovery. Subsequent functional training phases incorporated ambulation and stair negotiation exercises to restore fundamental mobility skills essential for community reintegration. After 27 days, as the patient’s lower limb strength gradually recovered (MRC score: 20 points), she began walking training on parallel bars. She was fitted with a knee brace to prevent collapse and a plastic short-leg orthosis on the ankle joint, with manual assistance to swing the limb. After 40 days, gait training with a walking aid commenced (MRC score: 34 points). The patient’s gait and strength improved gradually (MRC score: 44-50 points), and she was discharged 72 days after ventilator weaning. The entire protocol emphasized physiological stability while systematically challenging functional recovery through staged intensity progression. No hematoma, purpura, or infection recurrence occurred during her stay. The physical function at discharge is detailed in Table 1, and the MRC score and Barthel index trends are presented in Figure 3, the physical therapy program in Figure 4, and the change in blood parameters after extubation in Figure 5. Table 2 presents a comparison of our case with two previously reported cases that similarly exhibited profoundly low MRC scores (0-5 points) at the time of ICU-AW diagnosis [9,10]. The patient provided written informed consent for the publication of this case report.

**Table 2:** Comparison of the present case with two reported cases of ICU-AW diagnosed with MRC scores of 0-5 at onset This table compares cases with low MRC scores at ICU-AW diagnosis, demonstrating prolonged recovery periods across all instances MRC: Muscle Research Council; NMES: neuromuscular electrical stimulation; ICU-AW: intensive care unit-acquired weakness

Study	Primary disorder	Age and gender	Initial MRC assessment on diagnosis (point)	Mechanical ventilation (days)	Physical therapy program	MRC assessment and remarks
Tominaga et al. [9]	Acute abdomen and malignant lymphoma	60 years; woman	0	No detailed description(60-90 days)	Active rehabilitation (details not specified)	50 points at six months
						Tracheostomy
Chillura et al. [10]	Bronchopneumonia and acute kidney failure	56 years; man	5	No detailed description	Gait training	24 points at two months
					Tilt table	Tracheostomy
					NMES	Hemodialysis
					In-bed cycling	
Present case	Aspiration pneumonia and leukemia	Late 50s; woman	0	18 days	Standing	50 points at two months
					Gait training	
					Ergometer	
					Tilt table	

## Discussion

This case has two clinical implications. First, enhanced rehabilitation restores physical function in critically ill patients with ICU-AW. Second, physiotherapy is safe for immunosuppressed patients with prolonged thrombocytopenia during leukemia treatment.

Enhanced rehabilitation for severe ICU-AW

In previous studies, many patients retained modest residual strength (MRC score ~20) at extubation [11]. However, the patient was in a flaccid state, with an MRC score of 0. As scores of 36 or less imply severe ICU-AW [12], the current patient experienced significant muscle weakness during ventilator placement. In addition to analgesics and sedatives, various other medications, including steroids and vasopressors, were administered during mechanical ventilation. Steroids inhibit protein synthesis and accelerate muscle wasting [13], whereas sedatives induce mitochondrial dysfunction and reduce muscle metabolism, contributing to muscle weakness [14]. The patient received postremission therapy alongside frequent chemotherapy; prolonged myelosuppression and anemia-related effects may have weakened physical reserves, intensifying the case. Additionally, severe inflammation due to pneumonia likely raised cytokine levels that drive muscle degradation and oxidative stress, further accelerating myocyte damage. This extreme presentation may reflect the compounding effects of pancytopenia and severe pneumonia. Despite these overlapping risk factors, the patient achieved remarkable functional recovery, highlighting the impact of enhanced rehabilitation in hematological patients.　While outcomes vary significantly across cases due to heterogeneous backgrounds, this patient demonstrated more favorable muscle strength and ADL recovery trajectories compared to historical cases of severe ICU-AW with flaccid paralysis (MRC score: 0).

The rehabilitation guidelines for critically ill patients recommend enhanced rehabilitation after ICU discharge [3]; however, no standardized consensus exists on the optimal intensity and frequency. The rehabilitation intensity is determined based on an individualized assessment of each patient’s physiological reserve. In the current case, the duration (20-40 and 40-60 minutes) and frequency (five to seven times/week) of rehabilitation were increased. As upper limb function tended to recover more rapidly (Figure 3), occupational therapy was introduced to facilitate ADLs, including responding to nurse calls and eating, leading to early functional improvements. The patient’s hip flexion improved only slightly, even at 20 days of postextubation, which limited the physiotherapy options. Reduced exercise tolerance and low hemoglobin caused dyspnea, fatigue, and an increased heart rate, requiring frequent rest breaks. After 27 days, hip flexion and knee extension reached a “poor” level, enabling walking exercises with extensive assistance. In early physical therapy, neuromuscular electrical stimulation (NMES), in addition to a floor ergometer, is effective for preventing muscle weakness and improving hemodynamics [15]. However, NMES is generally contraindicated for patients with malignant tumors because of the risk of promoting tumor cell growth [16]. Recent studies have reported that combining resistance exercise with NMES improves muscle strength in patients with hematological malignancies [17]. Because the characteristics of cancer cells vary by tissue type and many aspects remain unknown, further case accumulation is needed.

Safety and effectiveness of physical therapy in patients with acute leukemia

In hematopoietic stem transplantation therapy, which is similar to acute leukemia due to long-term pancytopenia and severe immunodeficiency, early rehabilitation intervention is recommended to prevent decline in ADL and to maintain or improve quality of life [18]. Patients with low blood cell counts are particularly vulnerable to ADL deterioration and overall decline due to infectious and anemic symptoms associated with immunosuppression and physical activity limitations, such as bleeding tendencies [19]. These factors make it challenging to determine the appropriateness and intensity of rehabilitation interventions. Meanwhile, recent studies have reported both the safety and efficacy of exercise therapy, even in severely pancytopenic patients, highlighting the need for further case accumulation to strengthen clinical evidence [20]. Despite prolonged thrombocytopenia, the intervention carefully monitored subjective symptoms and objective signs (e.g., bleeding tendency). AML-specific factors like persistent cytopenia due to leukemia and its treatment may worsen ICU-AW through infections and inflammation. Adequate risk management enabled continuous intervention without serious adverse events, potentially facilitating recovery of muscle strength and ADLs.

## Conclusions

During leukemia treatment, the patient required mechanical ventilation and developed severe ICU-AW. Approximately two months of postextubation intervention led to ADL independence, and the patient was discharged with instructions for continued home training. These results suggest potential benefits of enhanced rehabilitation in similar cases, but larger studies are needed to generalize these findings.

## References

[1] Hermans G, Van den Berghe G (2015). Clinical review: intensive care unit acquired weakness. Crit Care.

[2] Hiser SL, Casey K, Nydahl P, Hodgson CL, Needham DM (2025). Intensive care unit acquired weakness and physical rehabilitation in the ICU. BMJ.

[3] Unoki T, Hayashida K, Kawai Y (2023). Japanese clinical practice guidelines for rehabilitation in critically ill patients 2023 (J-ReCIP 2023). J Intensive Care.

[4] Othman SY, Elbiaa MA, Mansour ER, El-Menshawy AM, Elsayed SM (2024). Effect of neuromuscular electrical stimulation and early physical activity on ICU-acquired weakness in mechanically ventilated patients: a randomized controlled trial. Nurs Crit Care.

[5] (2025). Ministry of Health, Labour and Welfare (MHLW), Japan. Part 2: results of physical condition survey. Annual National Health and Nutrition Survey Report. http://www.mhlw.go.jp/content/000711007.pdf.

[6] Morley JE, Abbatecola AM, Argiles JM (2011). Sarcopenia with limited mobility: an international consensus. J Am Med Dir Assoc.

[7] Hirasawa Y, Hasegawa T, Matsushita K, Yamazaki Y (2004). Isometric knee extension strength in healthy subjects. Phys Ther J.

[8] Cruz-Jentoft AJ, Bahat G, Bauer J (2019). Sarcopenia: revised European consensus on definition and diagnosis. Age Ageing.

[9] Tominaga T, Nonaka T, Takeshita H (2016). A case of intensive care unit-acquired weakness after emergency surgery for acute abdomen. Int J Surg Case Rep.

[10] Chillura A, Bramanti A, Tartamella F (2020). Advances in the rehabilitation of intensive care unit acquired weakness: a case report on the promising use of robotics and virtual reality coupled to physiotherapy. Medicine (Baltimore).

[11] Mehrholz J, Mückel S, Oehmichen F, Pohl M (2015). First results about recovery of walking function in patients with intensive care unit-acquired muscle weakness from the General Weakness Syndrome Therapy (GymNAST) cohort study. BMJ Open.

[12] Hermans G, Clerckx B, Vanhullebusch T (2012). Interobserver agreement of Medical Research Council sum-score and handgrip strength in the intensive care unit. Muscle Nerve.

[13] Yang T, Li Z, Jiang L, Xi X (2018). Corticosteroid use and intensive care unit-acquired weakness: a systematic review and meta-analysis. Crit Care.

[14] Okazaki K (2008). Evolution of propofol formulations and clinical challenges. J Jpn Soc Clin Anesth.

[15] Nakanishi N, Yoshihiro S, Kawamura Y (2023). Effect of neuromuscular electrical stimulation in patients with critical illness: an updated systematic review and meta-analysis of randomized controlled trials. Crit Care Med.

[16] Jin L, Hu B, Li Z, Li J, Gao Y, Wang Z, Hao J (2018). Synergistic effects of electrical stimulation and aligned nanofibrous microenvironment on growth behavior of mesenchymal stem cells. ACS Appl Mater Interfaces.

[17] Safran EE, Mutluay F, Uzay A (2022). Effects of neuromuscular electrical stimulation combined with resistance exercises on muscle strength in adult hematological cancer patients: a randomized controlled study. Leuk Res.

[18] Japan Cancer Rehabilitation Research Group (2015). Best Practices in Cancer Rehabilitation Care. Kanehara Publishing.

[19] Ishikawa A, Tsuji T (2016). The impact of rehabilitation on patients undergoing hematopoietic stem cell transplantation. J Hematop Cell Transplant.

[20] Elter T, Stipanov M, Heuser E, von Bergwelt-Baildon M, Bloch W, Hallek M, Baumann F (2009). Is physical exercise possible in patients with critical cytopenia undergoing intensive chemotherapy for acute leukaemia or aggressive lymphoma?. Int J Hematol.

